# Purification, characterization, and cloning of a novel pro-inflammatory secreted protein from *Staphylococcus aureus*

**DOI:** 10.1128/spectrum.02898-23

**Published:** 2023-11-08

**Authors:** Patrick M. Schlievert, Jacob D. Nelson, Samuel H. Kilgore, Lilliana Radoshevich, Aloysius J. Klingelhutz, Donald Y. M. Leung

**Affiliations:** 1 Department of Microbiology and Immunology, Carver College of Medicine, University of Iowa, Iowa City, Iowa, USA; 2 Department of Pediatrics, National Jewish Health, Denver, Colorado, USA; Emory University School of Medicine, Atlanta, Georgia, USA

**Keywords:** *Staphylococcus aureus*, virulence factors, chemokines, epithelial cells

## Abstract

*Staphylococcus aureus* is a human pathogen, causing infections ranging from benign skin and soft tissue infections to life-threatening pneumonia, sepsis/infective endocarditis, and toxic shock syndrome (TSS). The organisms cause infections through production of both cell-surface and secreted virulence factors. We focus primarily on secreted virulence factors, having done scans for such proteins for many years. We have purified and characterized TSS toxin-1; staphylococcal enterotoxin-like superantigens K, L, and Q; an operon of six serine proteases, epsilon cytotoxin; and enterotoxin-associated ampicillin resistance protein. The current study clones and characterizes another novel secreted factor that we tentatively refer to as methionine-rich protein (MRP) because its gene encodes a protein with ten methionine residues and eight in the small mature protein. The MRP gene encodes a protein of 199 amino acids with the first 28 being a signal peptide. The mature protein has 171 amino acids and has a molecular weight of approximately 18,800. MRP has an unusual isoelectric point (pI) for staphylococcal secreted virulence factors of approximately 5.3, whereas other secreted virulence factors have neutral or basic pIs. MRP was listed as a hypothetical protein in the NCBI database. We tested MRP for biological activities that characterize *S. aureus* and observed that the protein has no detectable superantigen, cytolytic (hemolytic), lipase, or protease activity. MRP induced chemokine (interleukin-8) production from human vaginal epithelial cells. This activity potentially facilitates *S. aureus* provoking harmful mucosal and skin inflammatory responses.

**IMPORTANCE:**

*Staphylococcus aureus* causes a myriad of human diseases, ranging from relatively mild soft tissue infections to highly fatal pneumonia, sepsis, and toxic shock syndrome. The organisms primarily cause diseases across mucosal and skin barriers. In order to facilitate penetration of barriers, *S. aureus* causes harmful inflammation by inducing chemokines from epithelial cells. We report the cloning and characterization of a novel secreted *S. aureus* protein that induces chemokine production from epithelial cells as its major demonstrable function. This secreted protein possibly helps *S. aureus* and its secreted proteins to penetrate host barriers.

## INTRODUCTION

*Staphylococcus aureus* is a ubiquitous bacterial pathogen, causing relatively benign to serious soft tissue infections, pneumonia, sepsis, infective endocarditis, osteomyelitis, and toxic shock syndrome (1 – 5). The organisms are also associated with numerous other conditions associated with immune dysfunction. These include atopic dermatitis (6), where *S. aureus* causes persistence of skin lesions; eczema herpeticum, which is a potentially severe form of atopic dermatitis (7); enterocolitis and Crohn’s Disease (8 – 12); bullous pemphigoid, which is a blistering autoimmune disease of the elderly (13); multiple sclerosis (14); rheumatoid arthritis (15); and a new superantigen-associated disease with a strong Th2 lymphocyte skew (16).

The organisms have a myriad of cell-surface and secreted virulence factors to facilitate disease causation. Among the secreted virulence factors are superantigens (2, 3), cytotoxins (hemolysins) (1, 17), proteases (1, 17), and lipase (glycerol ester hydrolase) (1, 17).

We have performed several large-scale general screens for novel secreted virulence factors by evaluating as many secreted proteins as possible from various strains of *S. aureus*. For example, we provided the first purification and characterization of toxic shock syndrome toxin-1 (TSST-1) (5); staphylococcal enterotoxin-like K, L, and Q (10 – 12); an operon of six serine proteases (18); a cytotoxin, which is referred to as epsilon cytotoxin (19); and an unpublished (by us) β-lactamase-like protein, which is referred to as enterotoxin-associated ampicillin resistance (EAR). We and Singh et al. (20) have been unable to find a phenotype for the EAR protein, even though its structure suggests that it is a β-lactamase.

We have now continued this screen with the identification of a novel secreted protein with 8 methionine amino acids in the mature protein and 10 in the translated protein. We tentatively refer to this protein as the methionine-rich protein (MRP). The current study provides the cloning and physicochemical and biological characterization of this novel protein. The MRP did not have demonstrable superantigen, cytotoxin (hemolysin), lipase (glycerol ester hydrolase), or protease activity. However, the protein had the ability to induce potentially harmful chemokine production by epithelial cells.

## RESULTS AND DISCUSSION

During our screens for secreted proteins from a variety of well-known *S. aureus* strains, we obtained a novel N-terminal sequence of a secreted protein after Edman degradation from strain RN6390 (RN450, NCTC8325). The N-terminal sequence of 10 amino acids was Aspartate-Threonine-Threonine-Serine-Methionine-Asparagine-Valine-Serine-Asparagine-Lysine.

**Fig 1:**
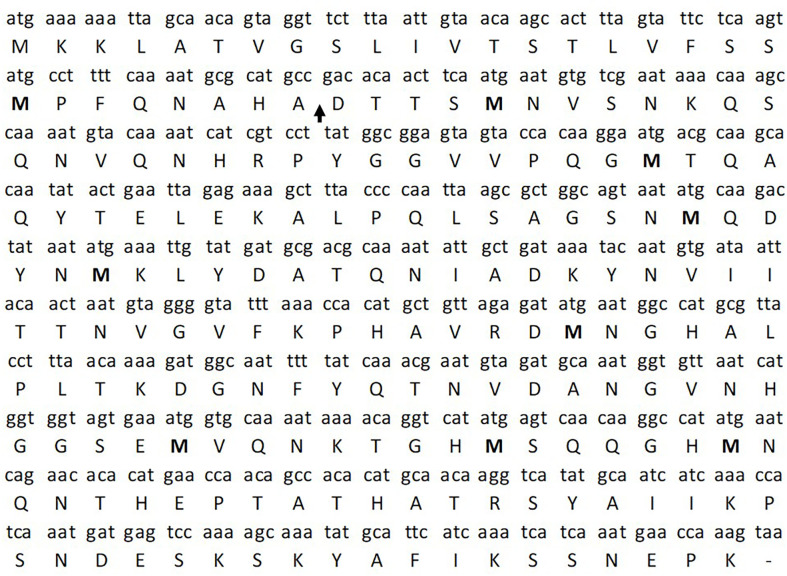
Nucleotide and translated protein sequences of the methionine-rich protein (MRP) from *Staphylococcus aureus*. The nucleotide sequence begins with a typical *atg* start codon and terminates with a *taa* stop codon. The single letter amino acid sequence is given below codons. The first 28 amino acids comprise a signal peptide with cleavage site indicated by the arrow. There were 10 methionine amino acids in the translated protein, with 8 in the mature protein. There were 199 total translated amino acids with 171 in the mature protein.

The examination of the protein database for *S. aureus* genes (https://blast.ncbi.nlm.nih.gov/Blast.cgi?PROGRAM=blastn&PAGE_TYPE=BlastSearch&LINK_LOC=blasthome) indicated that this protein was relatively methionine-rich, so we tentatively called the protein “methionine-rich protein” (MRP). There were eight methionine amino acids in the mature protein (Fig. 1). There were two additional methionine residues in the signal peptide for a total of 10 methionine amino acids in the entire translation product.

When we examined *S. aureus* strains, including RN6390, for the gene of the MRP, we located an open-reading frame of 597 nucleotides followed by a stop codon (Fig. 1). Thus, the entire translated protein was composed of 199 amino acids; the first 28 were a signal peptide, cleaved from the protein during secretion (Table 1). The mature protein contained 171 amino acids and had an estimated molecular weight of 18,800.

**TABLE 1:** Physicochemical and biological properties of the methionine-rich protein (MRP)

Property	Value
Number of amino acids in mature protein	171
Amino acid length of signal peptide	28
Total amino acids in the translated protein	199
Molecular weight of secreted protein	18,800
Isoelectric point of secreted protein	5.3
Superantigenicity	No
Protease	No
Lipase (glycerol ester hydrolase)	No
Pro-inflammatory chemokines from HVECs	Yes

We also used the DTU Health Tech website for signal peptide prediction (https://services.healthtech.dtu.dk/services/SignalP-6.0/). This website predicted the same signal peptide as was found by our comparison of the entire translated protein to the mature protein based on amino acid sequencing (first 10 amino acids).

Interestingly, MRP shared a significant sequence similarity to an *S. aureus* cell wall anchored protein called SasD (21). MRP and SasD are closely related proteins, differing in being secreted versus cell wall anchored and in several of the carboxy-terminal amino acids. As will be demonstrated in the current study and as suggested by Grousd et al. for SasD, both proteins appear to be important in pro-inflammatory responses.

Because the protein appeared to have numerous methionine amino acids, we compared examples of other secreted exotoxins from both *S. aureus* and *Streptococcus pyogenes* for percentages of methionine amino acids in the mature proteins (Table 2). MRP contained a higher percentage of methionine amino acids than all other secreted proteins tested, but the difference was not always significant by Fisher’s exact test.

**TABLE 2:** Methionine amino acids in mature secreted proteins of *S. aureus* and *Streptococcus pyogenes*

Secreted protein	Total amino acids	Methionines (%)	*P* value by Fisher’s exact
Methionine-rich protein (MRP)	171	4.7	**-**
Toxic shock syndrome toxin-1	194	1	0.05^*a*^
Staphylococcal enterotoxin C	239	1.7	NS^*b*^
Streptococcal pyrogenic exotoxin A	220	1.4	0.06
Streptococcal pyrogenic exotoxin C	208	1.4	0.07
Staphylococcal α-toxin	293	2.4	NS
Staphylococcal β-toxin	297	1	0.02

^*a*^ Compared to methionine-rich protein (MRP).

^*b*^ NS, not significant.

We performed a search for this protein in the NCBI database under *S. aureus* (https://blast.ncbi.nlm.nih.gov/Blast.cgi?PROGRAM=blastn&PAGE_TYPE=BlastSearch&LINK_LOC=blasthome). A protein with complete identity to MRP was an *S. aureus* hypothetical protein. Many, but not all *S. aureus* strains, tested to date contain MRP in their chromosomes. Some *S. aureus* strains lacked a translated protein with similarity to MRP. Thus, the MRP gene and the protein were variable traits in *S. aureus*, present in some strains but absent in others. As noted above, MRP is related to the cell wall anchored protein SasD (21).

**Fig 2:**
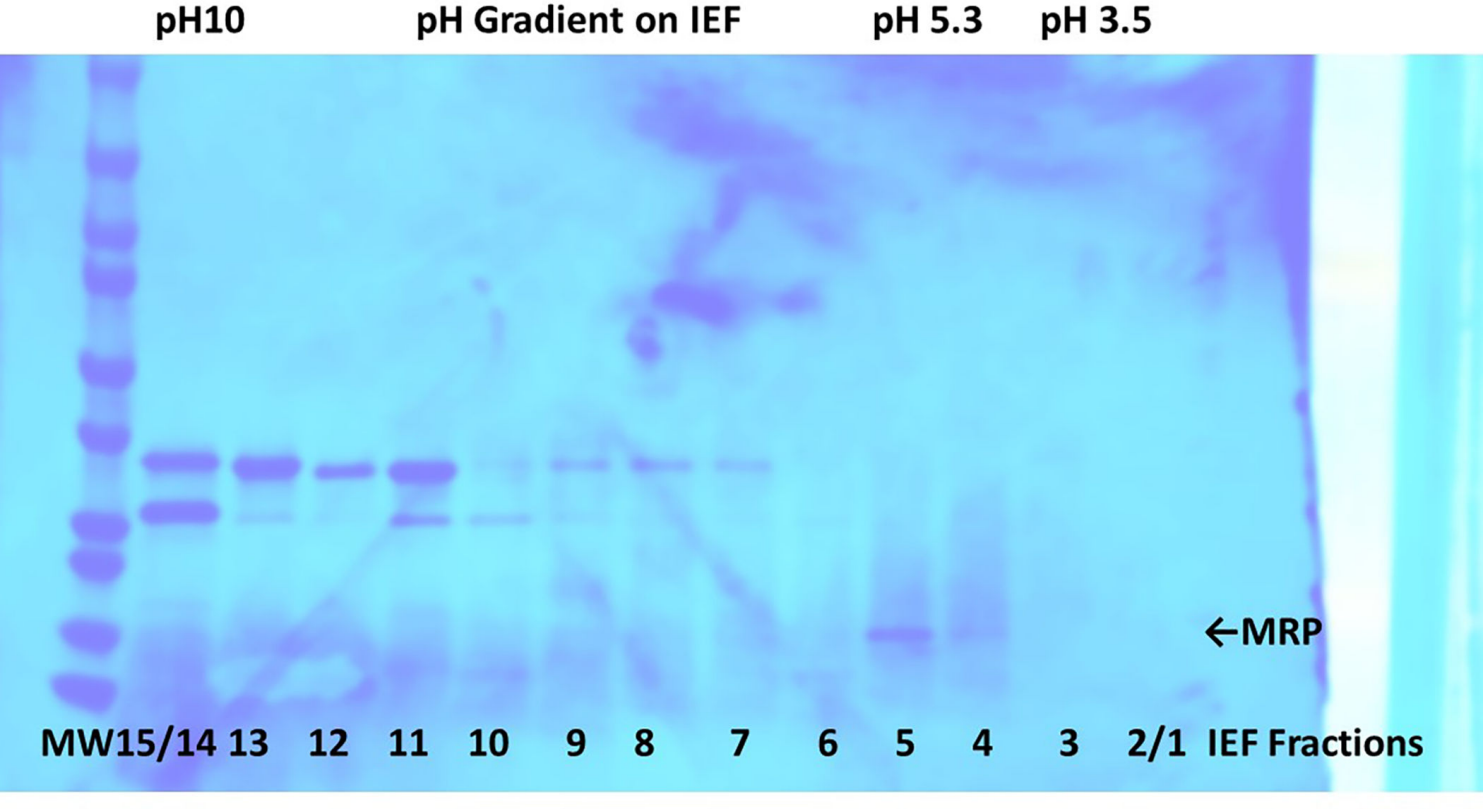
SDS-PAGE of IEF fractions for purification of the methionine-rich protein (MRP). For SDS-PAGE, approximately 1-cm fractions after preparative thin-layer IEF were collected, suspended in 10 mL of pyrogen-free distilled water, and 10 µL electrophoresed in the denaturing gel. Bands were visualized by staining with Coomassie brilliant blue R250, followed by destaining. Arrow points to MRP. The pH values of the acidic, basic, and MRP fractions are indicated.

We then purified MRP from *S. aureus* RN6390, as grown in 2,400 mL of Todd Hewitt broth, by precipitation from stationary phase cultures with 80% (final concentration) ethanol, resolubilization in pyrogen-free distilled water, followed by two rounds of preparative thin-layer isoelectric focusing (IEF) in pH gradients of 3.5 to 10. Fig. 2 shows sodium dodecyl sulfate polyacrylamide gel electrophoresis (SDS-PAGE) of 10 µL of each fraction after the second round of IEF. MRP from RN6390 focused in two 1-cm fractions (4 and 5) with isoelectric points of approximately 5.3. Most secreted proteins from *S. aureus* are neutral to basic, thus making purification of MRP relatively straightforward.

**Fig 3:**
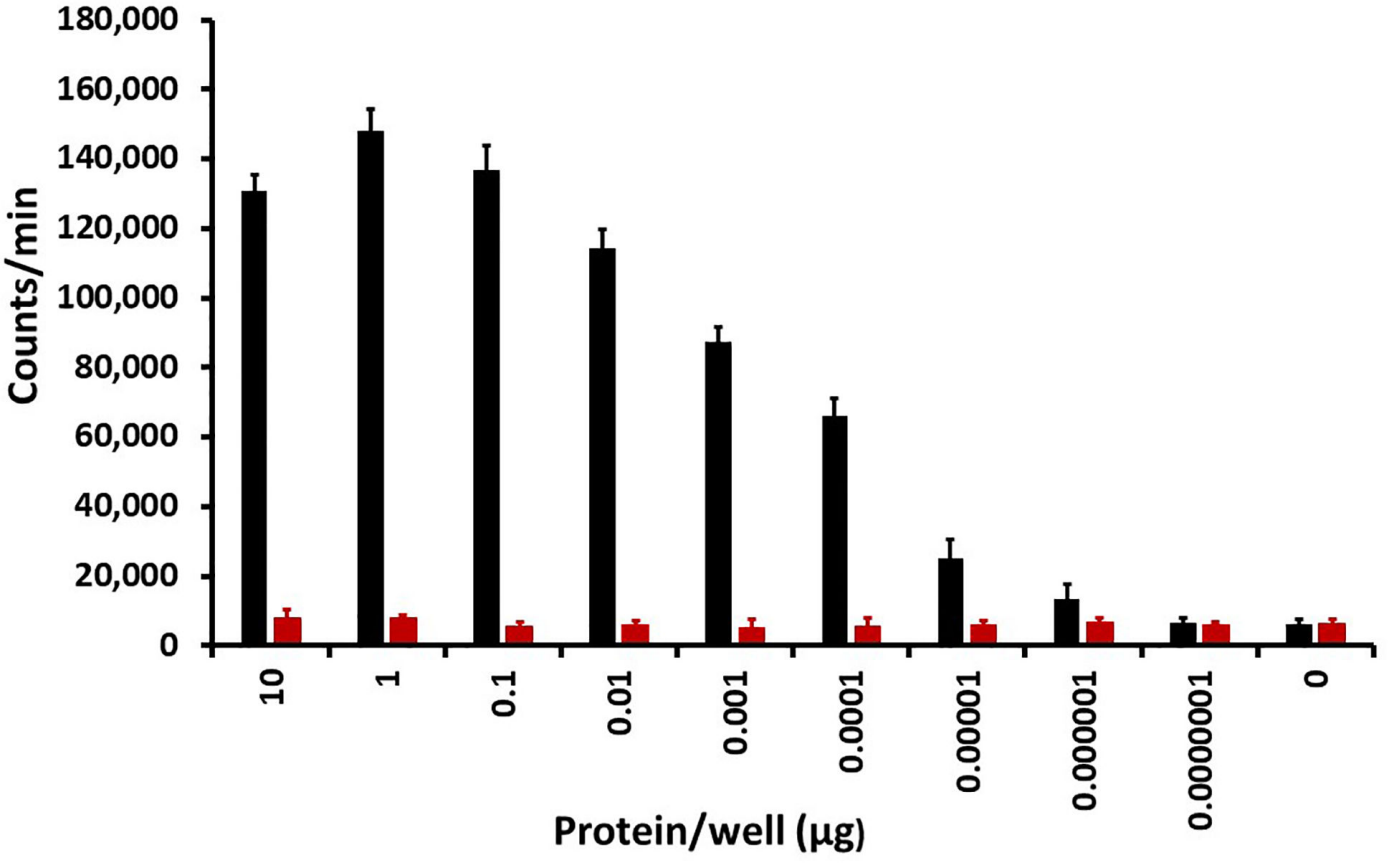
Superantigenicity assay of methionine-rich protein (red bars) compared to toxic shock syndrome toxin-1 (black bars), a known superantigen, ± standard deviations. Rabbit splenocytes were incubated for 3 days in 5% CO_2_ at 37°C with indicated concentrations of methionine-rich protein or toxic shock syndrome toxin-1 in quadruplicate wells. Then, 1 µCi of ^3^H-thymidine was added to all wells, and incubation was continued for another 24 h. DNA, indicative of lymphocyte proliferation, was harvested onto fiber glass filters, and counts per minute were determined with a scintillation counter.

We tested the protein, as purified from *S. aureus* RN6390, for superantigen activity. We used rabbit splenocytes for the proliferation assay compared to TSST-1 (Fig. 3). We note that *S. aureus* RN6390 is not known to secrete superantigen proteins, even though it does contain the gene for SE-like X. MRP was not related in amino acid sequence to SE-like X. MRP did not cause superantigen lymphocyte proliferation at any dose when compared to the known superantigen TSST-1 (Fig. 3). As expected, TSST-1 caused rabbit splenocyte proliferation at TSST-1 doses as low as 10^−6^ µg/well. MRP at the two highest doses caused a small amount of lymphocyte proliferation likely due to antigenic specific stimulation but not to superantigen stimulation.

**Fig 4:**
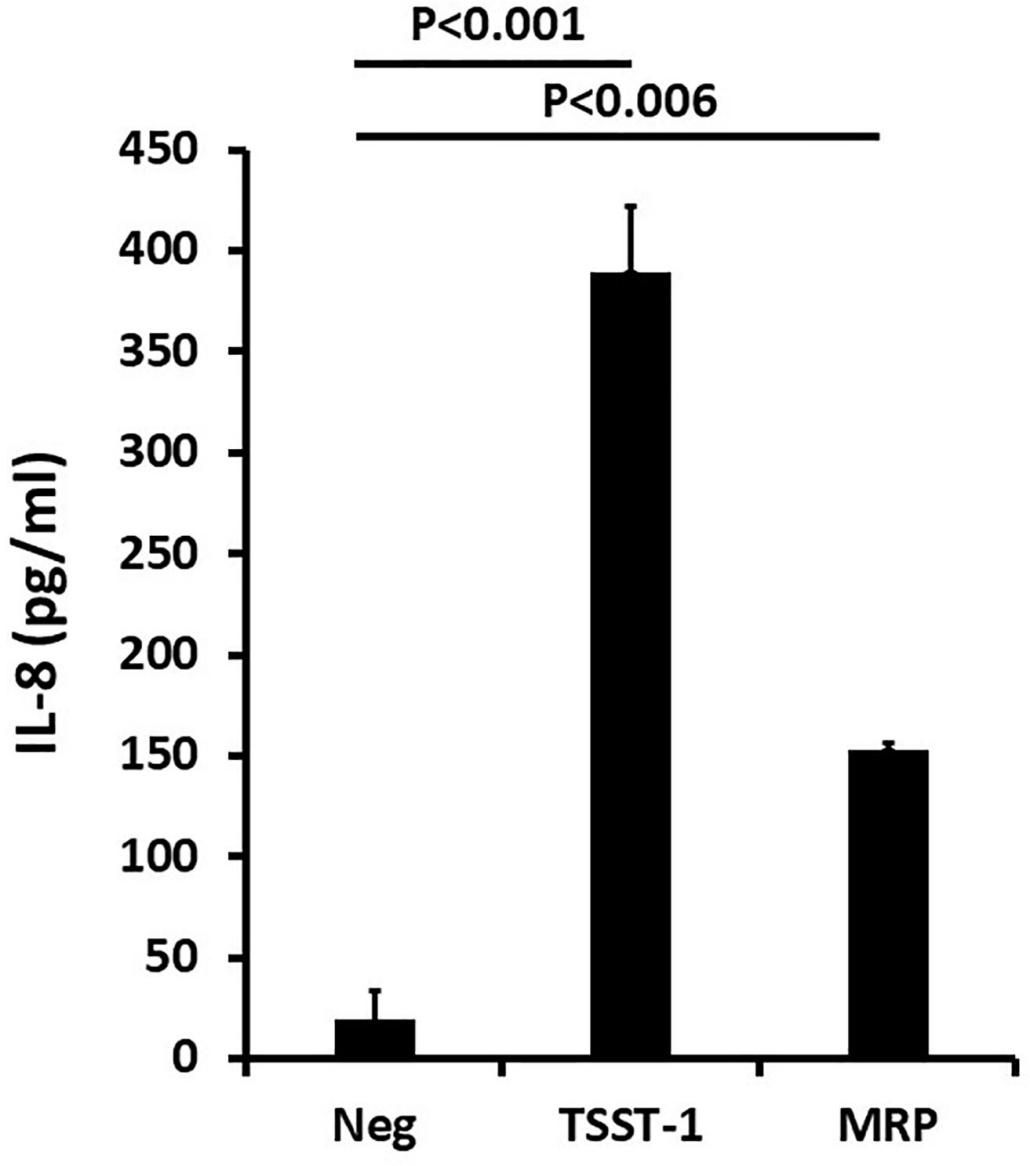
Induction of the chemokine IL-8 from human vaginal epithelial cells (HVECs) by both MRP and positive control toxic shock syndrome toxin-1 (TSST-1) in a 6-h assay. HVECs were incubated in triplicate with 50 µg of MRP or TSST-1 in keratinocyte serum-free medium for 7 h in a 5% CO_2_ incubator. Subsequently, IL-8 was determined in the supernates of the HVECs by use of a Quantikine ELISA kit from R&D systems (Minneapolis, MN).

MRP from RN6390 was next tested for cytotoxin (hemolysin), total protease, and lipase (glycerol ester hydrolase) activities. The protein lacked detectable activities. We also tested IEF fraction 5 of MRP from RN6390 for the ability to induce chemokine production (interleukin-8; IL-8) from human vaginal epithelial cells (HVECs) compared to the production by TSST-1 (Fig. 4). Both MRP and TSST-1 induced IL-8 production significantly above the negative control of HVECs only. This result gave us a phenotype for assays during cloning and purification from *Escherichia coli*. Additionally, this property was shared with the related cell wall anchored protein SasD (21).

We cloned the gene for MRP from RN6390 into *E. coli* DH5α. The insert gene for MRP was verified by nucleotide sequencing. Since the protein product of the cloned gene was secreted in *S. aureus*, we reasoned that the protein, as expressed in *E. coli*, would be contained in the periplasm. We thus grew the *E. coli* clone plus separately *E. coli* with the empty vector plasmid, each in 5 mL of Luria Bertani broth with antibiotic. Subsequently, both organisms were treated with lysozyme-EDTA for 30 min and then heat (100°C, 2 min) to lyse the *E. coli* cells. The cell debris was removed by centrifugation, and the supernatant fluids were tested preliminarily for biological activity [ability to induce chemokine (IL-8) production from HVECs]. The reason that we focused on this biological activity, as noted above, was because MRP was a novel protein with no similarity to *S. aureus* proteases, cytotoxins (hemolysins), lipase (glycerol ester hydrolase), or superantigens. Thus, knowing that *S. aureus* can be highly pro-inflammatory with chemokine production by epithelial cells (22), we examined the supernatant fluids for induction of IL-8 production, a chemokine that is known from previous studies to be a marker for inflammatory responses from HVECs. The clone supernatant fluid induced IL-8 production, which was significantly higher than supernatant fluid from the empty vector control.

It is important to note that the HVEC line used in our studies lacks Toll-like receptor 4 and is not responsive in IL-8 production to *Enterobacteriaceae* lipopolysaccharide.

We then grew the *E. coli* clone in 2,400 mL of Todd Hewitt broth containing antibiotic until stationary phase. Then, the cells were treated with lysozyme-EDTA and heat as before, the bacterial cell debris was removed by centrifugation, and the supernatant fluids were subjected to two rounds of preparative thin-layer isoelectric focusing (IEF). This same technique, thin-layer IEF, was the method (23) we originally used on concentrated supernatant fluids from *S. aureus*, which led to detection of MRP and many other secreted proteins in our initial screens. Thus, we believed that we would be able to locate the protein from the two rounds of preparative thin-layer IEF.

**Fig 5:**
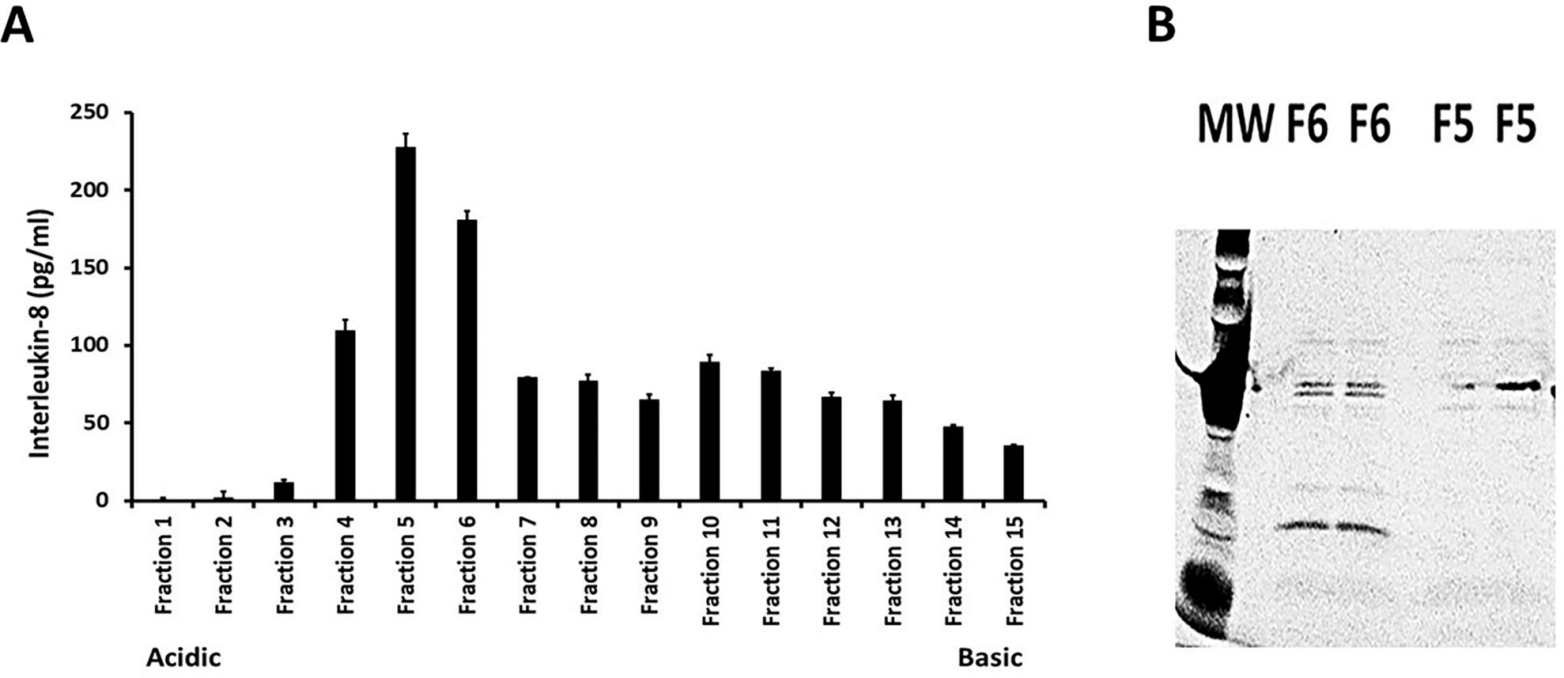
Preparative thin-layer isoelectric focusing (**A**) of the methionine-rich protein, cloned into *E. coli* DH5α, with gene derived from *Staphylococcus aureus* strain RN6390. Each 1-cm fraction was separated from the matrix gel and tested for stimulation of IL-8 production from human vaginal epithelial cells after 6 h of incubation. Fractions 5 and 6 were evaluated for biochemical (5B; SDS-PAGE) and biological activities due to their induction of high-level IL-8 production; the amount of IL-8 isolated was comparable to that observed for toxic shock syndrome toxin-1, a known agent to induce chemokine production from these cells. The isoelectric point of fractions 5 and 6 were 5.2 and 5.4, respectively.

Our available screen for MRP biological activities was the induction of IL-8 production from HVECs. Thus, we collected 15 one-centimeter fractions from the second preparative IEF, and these were tested individually in triplicate for induction of IL-8 from HVECs after 6 h of exposure. Fractions 5 and 6 (isoelectric points 5.2 and 5.4) induced the highest IL-8 production (Fig. 5A). The proteins from these two fractions were eluted and dialyzed for 4 days to remove ampholytes. The protein concentrations were determined (18 mg and 11 mg amounts from fractions 5 and 6, respectively, from the 2,400-mL original culture). These corresponded well to the same estimated amounts of the MRP as purified originally from *S. aureus* RN6390. When an additional preparation of MRP was similarly evaluated using the same procedures, MRP mapped to IEF fractions 6 and 7, close to the prior assay. By SDS-PAGE, the purified protein was determined to have a molecular weight of approximately 19,000 close to the estimated weight of MRP from the gene and protein sequence as shown in Fig. 1.

The isoelectric points (pIs) of 5.2 and 5.4 are unusual for secreted protein virulence factors of *S. aureus*. Nearly, all are neutral (e.g., staphylococcal enterotoxin A, TSST-1, and staphylococcal α-toxin) or strongly basic (e.g., staphylococcal enterotoxins B–E) (2 – 4). The significance of the acidic pI is unknown.

The two IEF fractions were lyophilized and suspended to 250 µg/mL for bioassays. Twenty microliters of each fraction (50 µg) was tested for hemolytic activity against rabbit erythrocytes, for protease activity by a colorimetric assay for general proteases, and for lipase activity by hydrolysis of tributyrin in microscope slide assay. Neither fraction had significant activity in any of the tests.

Finally, purity studies of MRP fractions 4 and 5 from *E. coli* were performed by assessing homogeneity by SDS-PAGE (Fig. 5B). Fraction 4, which was less able to induce IL-8, was contaminated with several unknown proteins. However, fraction 5, which induced greater IL-8 from HVECs, had only minor contaminates.

For our studies of MRP, we conclude that the major biological activity of this novel protein was to be pro-inflammatory to HVECs.

## MATERIALS AND METHODS

### Bacteria

RN6390 was used as the source *S. aureus* strain for this broad screen for secreted virulence factors. This strain is derived from NCTC8325 and is related to RN450. The initial screen was performed by culturing RN6390 in 2,400 mL of Todd Hewitt broth dialysate until stationary phase at 37°C with 200 revolutions per minute shaking. The Todd Hewitt dialysate was prepared by making a 10× concentrated suspension of Todd Hewitt (Difco Laboratories, Detroit, MI) broth and dialyzing this for 4 days against 2,400 mL of pyrogen-free distilled water at 4°C without shaking. The dialysis tubing had a molecular weight cut-off of 12,000–14,000. The pyrogen-free dialysate was then sterilized and inoculated with RN6390. Regular Todd Hewitt broth was prepared according to the manufacturer’s (Difco) instructions.

After growth of RN6390 to stationary phase, the culture fluid was treated with 4 volumes of absolute ethanol to precipitate proteins, generally of >10,000 molecular weight. After 2 days at 4°C, the precipitated proteins were collected by centrifugation (4,000 × *g* for 15 min). After drying off the residual ethanol, the precipitate was suspended in 100 mL of pyrogen-free distilled water and centrifuged at 10,000 *× g* for 30 min. The supernatant fluid was collected and dialyzed for 24 h at 4°C without shaking against 2 L of pyrogen-free distilled water to remove excess salts (dialysis tubing with a 12,000–14,000 molecular weight cut-off). The contents of the dialysis bag were subjected to two rounds of preparative thin-layer isoelectric focusing (IEF) (both rounds with pH gradients of 3–10). After the first round of IEF, the extreme acidic brown residual media proteins were removed and the remainder re-focused in a new pH gradient of 3–10. Subsequently, a piece of Whatman number 1 filter paper (Sigma-Aldrich, St. Louis, MO) was laid over the completed focusing run for 30 s to absorb a portion of the focused proteins. The filter paper was carefully removed, washed once in ethanol-water-acetic acid (8:3:1), and stained with Coomassie brilliant blue R250 [Sigma-Aldrich; 0.25 g/100 mL methanol:water:acetic acid (ratio = 45:45:10)]. Then, the filter paper was destained with ethanol-water-acetic acid until protein bands became visible. The visible bands on the filter paper were used to guide scraping focused proteins from the granular inert matrix gel [Sephadex G75 superfine (Sigma-Aldrich) washed with pyrogen-free distilled water (6 L/100 of Sephadex beads and then beads collapsed with 4 L of absolute ethanol)]. Proteins were removed from the IEF gel by elution through glass fiber filters, and potentially novel proteins including the MRP were dialyzed for 4 days versus 1 L of pyrogen-free distilled water; protein concentration was then determined (Bio-Rad Protein Assay, Bio-Rad, Hercules, CA), and samples were lyophilized. The N-terminal 10-amino-acid sequence of MRP was determined at the Mayo Clinic (Rochester, MN) after Edman degradation. The MRP from RN6390 was tested for biological activities common to *S. aureus*: superantigen (rabbit splenocyte proliferation), cytotoxin (lysis of rabbit erythrocytes), lipase (glycerol ester hydrolase; hydrolysis of tributyrin), and protease (colorimetric assay). The assays used are described in detail below. RN6390 is known to have the gene for the superantigen SE-like X, but the strain does not produce detectable superantigen protein.

### Superantigen test

Animal use was in accordance with an approved protocol from the University of Minnesota Institutional Animal Care and Use Committee. MRP from *S. aureus* 6390 was tested for superantigenicity, compared to TSST-1, by use of rabbit splenocytes in a 4-day assay (24). Briefly, 2 × 10^5^ rabbit splenocytes/200 µL RPMI 1640 media were placed in quadruplicate in wells of 96-well tissue culture plates. MRP was added to the wells at concentrations of 10, 1, 10^−1^, 10^−2^, 10^−3^, 10^−4^, 10^−5^, 10^−6^, 10^−7^, and 0 µg/20 µL/well. Identical wells contained the same concentrations of TSST-1, a known superantigen. Wells were incubated at 37°C for 3 days, and then 1 µCi ^3^H-thymidine was added to all wells. The plates were incubated another 24 h, and DNA was collected on glass fiber filters as a measure of lymphocyte proliferation.

### Cloning of the MRP gene

Because of the potential problem that the measured biological activities could be complicated by staphylococcal contaminants, the novel MRP was cloned into *E. coli*, and active fractions from preparative IEF were re-tested for comparable biological activities.

*E. coli* DH5α was used as the host strain for cloning the MRP. The vector plasmid was pCE104 (25). With the use of the N-terminus of MRP, it was possible to find its gene in the NCBI database. Primers that encompassed the putative signal peptide and upstream possible promoters and the downstream putative termination sites were prepared. The primers were used to prepare *mrp* DNA by polymerase chain reaction for cloning. The gene was cloned into the multiple cloning site of pCE104 with blue-white screening (25). Verification of cloning was performed by isolating the plasmid with insert from a clone and sequencing *mrp*.

### Other assays of MRP

SDS-PAGE was performed with the method of Laemmli (26).

MRP as present in the NCBI database site was listed as a hypothetical protein. We first cultured 5 mL of *E. coli* DH5α with the *mrp* insert and with the empty vector plasmid. Culturing was in Luria Bertani broths with shaking (200 revolutions per minute) at 37°C. Once stationary phase was achieved, the *E. coli* cells were collected by centrifugation (4,000 × *g* for 15 min). The cells were treated with lysozyme (25 µg/mL) and EDTA [Tris-EDTA buffer, pH 8.0 (0.02 M Tris and 0.01 M EDTA)] for 30 min at 37°C and then heat (100°C, 2 min). The lysed cells were centrifuged (14,000 × *g* for 5 min) and supernatant fluids were collected.

The supernatant fluids from both the clone of *mrp* and the empty vector control were evaluated for lipase, protease, and hemolytic activities (measure of cytotoxic activity). For the lipase assay, tributyrin (Sigma-Aldrich) was suspended (80 µL/4.5 mL) in 0.85% agarose in phosphate-buffered saline [PBS; 0.005 M NaPO_4_ (pH 7.2) and 0.15 M NaCl]. Microscope slides were coated with 4.5 mL of this turbid suspension and agarose was allowed to solidify. Subsequently 4-mm wells were punched in the agarose, and 20 µL of supernatant fluids was added to the wells. The plates were incubated for 24 h at 37°C and then examined for clearing due to tributyrin hydrolysis. Supernatant fluid from *S. aureus* MN8 (20 µL) was used as the positive control for lipase activity.

For protease activity, the supernatant fluids were assayed exactly as described in a kit purchased from Thermo Scientific (colorimetric protease assay kit product number 23263).

For hemolytic activity, 20 µL of supernatant fluids was added to a 4-mm well punched in rabbit blood agar plates (Remel; Thermo Scientific) (27). Positive control for hemolysis was supernatant fluid from *S. aureus* MNPE. Plates were incubated at 37°C for 24 h, and then zones of hemolysis were measured.

The supernatant fluids were assayed for the ability to induce chemokine production by isolated HVECs (28). The representative chemokine was IL-8, measured after 6 h of incubation at 37°C in a keratinocyte serum-free medium. Quantikine kits were purchased from R&D Systems (Minneapolis, MN) for IL-8 ELISA. Since the only assay that gave a phenotype was the production of IL-8 from HVECs, this was used to follow MRP during purification.

### Final purification of MRP

MRP was purified from 2,400 mL of *E. coli* cells after culture in Todd Hewitt broths with ampicillin (50 µg/mL) and shaking (200 revolutions per minute), 37°C until stationary phase. The *E. coli* cells were collected by centrifugation (4,000 × *g* for 15 min) and lysed with lysozyme-TE (pH 8.0) and EDTA and heat as above. The lysates were centrifuged (10,000 × *g* for 30 min) and supernatant fluids were subjected to two rounds of preparative thin-layer IEF, both in 3–10 gradients. The most acidic, brown-yellow residual media proteins were removed after the first round, and the remaining proteins re-focused in the second round. Subsequently, fifteen 1-cm fractions were collected from the thin-layer plate, the gel was suspended in 10 mL of pyrogen-free distilled water, the matrix gel was settled out, and supernatant fluids were assayed for IL-8 production from HVECs.

The fractions with the greatest ability to induce IL-8 (fractions 5 and 6 in first assay and fractions 6 and 7 in the second assay) were eluted from the matrix gel, their pHs were determined, and then they were dialyzed for 4 days against 1 L of pyrogen-free distilled water. Subsequently, the two fractions were assayed for protein concentration and lyophilized separately. The two fractions were resuspended to 250 µg/mL in PBS and assayed for reproducing hemolytic, lipase, and protease activities as above with use of 50 µg MRP/20 µL.

### Statistical analysis

Differences in means were tested by Student’s *t* test analysis of unpaired, normally distributed data where appropriate. In assays for differences in methionine content, Fisher’s exact test was used.
